# Prodigiosin Enhanced TMZ Chemosensitivity by Suppressing Focal Adhesion and Inhibiting Autophagy in Glioblastoma Cells

**DOI:** 10.3390/biom16070977

**Published:** 2026-07-03

**Authors:** Shihui Dai, Xin Liu, Xiangyu Jin, Shaoming Mo, Li Li, Chuan Wang, Yaomei Tian

**Affiliations:** School of Food and Liquor Engineering, Sichuan University of Science & Engineering, No. 519, Huixing Road, Ziliujing District, Zigong 643000, China

**Keywords:** glioblastoma, prodigiosin, temozolomide, combinatorial strategy

## Abstract

Glioblastoma (GBM) remains a lethal brain tumor with poor prognosis and limited therapeutic efficacy from temozolomide (TMZ) treatment. Prodigiosin (PG), a bioactive secondary metabolite, has demonstrated anti-tumor activity across a broad spectrum of tumors. This study aims to investigate the therapeutic potential and mechanism of PG combined with TMZ in treating GBM. The results demonstrated that the combination of PG and TMZ synergistically inhibited GBM cell proliferation, triggered apoptosis, and suppressed migration and invasion. Transcriptomic analysis revealed downregulation of focal adhesion and related signaling pathways. Functionally, the combination therapy reduced focal adhesion numbers and AKT phosphorylation. Co-treatment with PG and TMZ impaired autophagic flux, evidenced by LC3-II and p62 accumulation. Furthermore, the anti-proliferative effect and the accumulation of LC3-II and P62 by the combination therapy were enhanced by the autophagy inhibitor chloroquine (CQ) but not reversed by the autophagy activator rapamycin (Rapa), confirming autophagy inhibition as a key mechanism. In conclusion, PG sensitized GBM cells to TMZ by impairing autophagy and focal adhesion signaling, providing a preclinical rationale for the combinatorial strategy.

## 1. Introduction

Glioblastoma (GBM) is commonly observed as the most prevalent and aggressive malignant brain tumor in adults, characterized by rapid progression, therapeutic resistance and poor prognosis [1]. Surgical resection followed by radiotherapy and/or chemotherapy constitutes the standard treatment for GBM. However, GBM has a poor prognosis and high recurrence mainly due to the high rates of malignancy and insensitivity to chemoradiotherapy. According to studies, the 5-year overall survival rate is less than 10%, with a median survival time of less than 15 months [2]. Temozolomide (TMZ), an alkylating agent with rapid absorption and increasing penetration through the blood–brain barrier [3], remains one of the only drugs approved for first-line treatment of glioblastoma. The standard regimen of post-operative radiotherapy combined with TMZ significantly improves survival outcomes over radiotherapy alone: median overall survival increases by 2.5 months (from 12.1 to 14.6 months), with the two-year survival rate reaching 26% [4]. Nevertheless, TMZ efficacy is significantly limited by acquired resistance which occurs in roughly 50% of patients. The underlying mechanisms are multifactorial, encompassing overactivation of DNA repair pathways, impaired intracellular TMZ accumulation, and Heterogeneous tumor microenvironment such as glioma stem cells, hypoxia, and protective autophagy [5].

Autophagy is a fundamental cellular process essential for maintaining homeostasis and ensuring cell survival. Autophagy can be activated in response to stressors including nutrients, growth factors, energy levels, oxygen levels, oxidative stress, ER stress, and pathogen infection, and then eliminate or degrade DNA debris, protein aggregates and other damaged organelles [6]. Th dysregulation of this process in tumor cells is closely linked to tumor advancement, metastasis, and drug resistance [7]. Tumor necrosis and acidic stress within the GBM microenvironment can activate autophagy, thereby promoting tumor invasion and conferring resistance to radiotherapy and chemotherapy [8,9]. Moreover, the autophagy pathway can be triggered by TMZ treatment in glioma cells [10] by multiple-signaling pathways including the activation of the ATM-AMPK-ULK1 axis [11], the production of a cytoprotective adenosine triphosphate surge [12], the modulation of NRF2 and MAPK-signaling activation via Polyphyllin I [13], and the dependence on the induction of O^6^-methylguanine [14] and TTK Protein Kinase [15]. Consequently, application of autophagy inhibitors represents an effective strategy for potentiating TMZ cytotoxicity in GBM cells. In preclinical studies, several agents have been shown to enhance the efficacy of TMZ by inhibiting autophagy, including Chloroquine [16], daurisoline [17], Biochanin A [18], Hispidulin [19], Bortezomib [20], and so on.

Prodigiosin (PG), a bioactive secondary metabolite primarily produced by *Serratia marcescens* and other microorganisms, demonstrates a wide range of promising therapeutic activities [21]. In recent years, PG has garnered significant research attention for its potential application in tumor therapy. PG has been shown to exhibit anticancer effects against various tumors, including papillary thyroid cancer [22], triple negative breast cancer [23], myeloma [24], colon Cancer [25], glioblastoma [26], and so on. PG also demonstrates the potential to sensitize tumor cells to chemotherapeutic agents such as Cisplatin [27] and 5-Fluorouracil (5-Fu) [28], thereby achieving a synergistic inhibitory effect on tumor growth. Cheng et al. reported that PG exerted the antitumor effect in glioblastoma by activating the JNK pathway while inhibiting the AKT/mTOR pathway, causing autophagic cell death [26]. Zhao et al. demonstrated that PG functioned as a novel autophagy inhibitor in colorectal cancer cells by blocking late-stage autophagosome–lysosome fusion and then sensitized colorectal cancer cells to 5-Fu [28,29]. In our previous study, PG was prepared from *Serratia marcescens* Ka3 and showed anti-tumor activity against human lung carcinoma A549 cells, human colon adenocarcinoma HT29 cells and human gastric adenocarcinoma SGC7901 [30]. Regarding the established role of PG in modulating autophagy pathways, we hypothesize that PG has the potential to sensitize GBM cells to TMZ.

In the study, we aim to explore the therapeutic potential and mechanism of combining PG with TMZ against GBM. The results revealed that PG can increase the chemosensitivity of GBM cells to TMZ by significantly inhibiting GBM cell viability, inducing apoptosis, suppressing focal adhesion signaling and impairing autophagic flux, thereby providing a novel and preclinical rationale for glioma treatment.

## 2. Methods

### 2.1. Cell Line and Reagents

Human GBM cell lines T98G were obtained from Procell (Wuhan, China) and A172 from the Shanghai Institute of Cell Biology, Chinese Academy of Sciences (Shanghai, China). Both cells were cultured with DMEM medium containing 10% FBS and 1% antibiotics. Temozolomide (TMZ) were purchased from MCE (HY-17364, Monmouth Junction, NJ, USA). Prodigiosin (PG) was prepared and purified by our laboratory following the protocol described in our previous study [30], the purity of the compound was confirmed to be >98% by HPLC analysis.

### 2.2. Cell Viability and Proliferation Assay

Cells were seeded at 5000 per well in 96-well plates and incubated overnight at 37 °C with 5% CO_2_. The next day, cells were treated with different concentrations of TMZ either alone or in combination with PG. After 48 h, the culture media was removed from the wells and the CCK-8 reagent was added to each well according to the instructions (MC0301-500, KERMEY, Zhengzhou, China). The plate was then returned to the incubator for an hour at 37 °C. After the incubation, the absorbances at 450 nm were measured on a microplate reader (BIORAD, Hercules, CA, USA). Cell viability was calculated as a percentage relative to the untreated cells based on absorbance readings.

For the inhibition assays, T98G and A172 cells were treated with rapamycin (20 μM; MCE, Monmouth Junction, NJ, USA), chloroquine (30 μM; Sigma, St. Louis, MO, USA), or escalating concentrations of Bafilomycin A1 (50, 100, or 250 nM; AmBeed, Arlington Heights, IL, USA). All three inhibitors were co-treated with TMZ and TP, followed by 48 h of incubation prior to subsequent CCK-8 assays.

### 2.3. EDU Assay

Cells were seeded at 70,000 per well in 24-well plates and incubated overnight at 37 °C with 5% CO_2_. The following day, cells were co-treated with PG (0.5 μg/mL) or TMZ (800 μM for T98G and 600 μM for A172, respectively) for 48 h. After treatment, cells were pulsed with 10 μM EdU reagent (Beyotime, Shanghai, China) at 37 °C for 2 h, fixed with 4% paraformaldehyde for 15 min at room temperature, incubated with 0.5% (*v*/*v*) Triton X-100 for 15 min at room temperature, and then added to Click Additive Solution at room temperature for 30 min under light-protected conditions. After washing with PBS, nuclei were counterstained with DAPI. Observation and imaging were performed using the DeltaVision Ultra high-resolution live cell imaging system (Leica, Wetzlar, Hesse, Germany). EdU-positive cells exhibited red fluorescence, while the nuclei displayed blue fluorescence. The number of EdU-positive cells and the total cell count were analyzed using ImageJ-Fiji software, and the EdU positivity rate was calculated to evaluate cell proliferation capacity.

### 2.4. PI Staning

T98G and A172 cells were respectively seeded at 150,000 per well in 6-well plates and incubated overnight at 37 °C with 5% CO_2_. The following day, cells were co-treated with PG (0.5 μg/mL) or TMZ (800 μM for T98G and 600 μM for A172, respectively) for 48 h. After treatment, cells were pulsed with 2 μg/mL propidium iodide reagent (PI, biosharp, Hefei, China) at 37 °C for 5 min, and then immediately observed under a fluorescence microscope (Nikon, Tokyo, Japan).

### 2.5. Cell Apoptosis Analysis

Annexin V-PE/7-AAD (YEASEN, Shanghai, China) was employed to assess apoptosis rates. T98G and A172 cells were respectively inoculated into 6-well plates at a density of 1.5 × 10^5^ cells per well and incubated overnight at 37 °C with 5% CO_2_. The following day, cells were co-treated with PG (0.5 μg/mL) or TMZ (800 μM for T98G and 600 μM for A172, respectively) for 48 h. After treatment, all cells were collected and centrifuged at 300× *g* for 5 min at 4 °C, followed by two washes with PBS. The pellet was then resuspended with 100 μL 1× Binding Buffer which was added with 5 μL Annexin V/PE and 10 μL 7-AAD at room temperature for 15 min under light-protected conditions. After adding with 400 μL 1× Binding Buffer, cells were analyzed using a flow cytometer (Agilent, Santa Clara, CA, USA).

### 2.6. Wound Healing Assay

T98G (2.5 × 10^5^ cells per well) and A172 (2.0 × 10^5^ cells per well) cells were respectively seeded into 12-well plates and incubated overnight at 37 °C with 5% CO_2_. The following day, after creating a straight wound in each well using a pipette tip, detached cells were gently washed away with a DMEM medium. The cells were then cultured with fresh DMEM supplemented with 1% FBS and 1% antibiotics, and co-treated with PG (0.5 μg/mL) or TMZ (800 μM for T98G and 600 μM for A172, respectively) for 24 h. At 0 h, images of the scratched area were captured under a microscope (Nikon, Japan) at fixed positions. The cells were then returned to the incubator at 37 °C with 5% CO_2_ for 24 h. Images were taken again at the same positions. The area of the scratch at each time point was measured using ImageJ software to calculate the migration rate.

### 2.7. Cell Migration Assay

The 2 × 10^4^ cells were suspended in 200 μL DMEM medium and seeded into the 24-well plate inserts (8.0 μm Pore size, NEST, Wuxi, China), while the lower 24-well plate contained 600 μL DMEM medium supplemented with 10% FBS and 1% antibiotics. The cells in the insert were co-treated with PG (0.5 μg/mL) or TMZ (800 μM for T98G and 600 μM for A172, respectively) for 24 h. Non-migrated cells in the insert were carefully removed. The migrated cells at the bottom of the insert were fixed with methanol for 10 min, stained with 0.5% crystal violet (YEASEN, China) for 10 min, washed with PBS, and finally were observed under an inverted microscope (Nikon, Japan).

### 2.8. Western Blot Analysis

Treated cells were harvested and then lysed on ice for 30 min with RIPA Lysis buffer (Beyotime, China). The collected cell pellets were subjected to 25% ultrasonic disruption and then centrifuged at 12,000 rpm for 10 min at 4 °C. Protein concentrations were determined using a BCA protein assay kit (Beyotime, China). Protein samples were separated by SDS-PAGE and electro-transferred to polyvinylidene fuoride membranes (Millipore, Burlington, MA, USA). The membranes were blocked with 5% non-fat milk prepared in PBST at room temperature for 1 h, and then incubated overnight at 4 °C with specific primary antibodies. On the following day, the membranes were incubated with the corresponding secondary antibodies at room temperature for 1 h. Protein bands were visualized using an enhanced chemiluminescence (ECL) detection reagent (Biosharp, Hefei, China) and observed using an imaging system (e-BLOT, Shanghai, China). Antibodies against PARP (9542T, 1:1000, cell signaling technology, Danvers, Massachusetts, USA), Caspase-3 (S966ZS, 1:1000, cell signaling technology, Danvers, Massachusetts, USA), GAPDH (ET1601-4, 1:3000, HUABIO, Hangzhou, China), N-cadherin (13116T, 1:1000, cell signaling technology, Danvers, MA, USA), pAKT (ET1607-73, 1:2000, HUABIO, Hangzhou, China), AKT(ET1609-51, 1:2000, HUABIO, Hangzhou, China), LC3B (ET1701-65, 1:2000, Abmart, Shanghai, China), P62 (T55546F, 1:5000, Abmart, Shanghai China), and Horseradish Peroxidase (HRP)-conjugated goat anti-Rabbit IgG secondary antibody (HA1001, 1:50,000, HUABIO, Hangzhou, China) were used in the present study.

### 2.9. Fluorescence Microscopy

Glass coverslips (NEST, China) were placed into 12-well plates. T98G and A172 cells were respectively inoculated into 12-well plates at a density of 7.0 × 10^4^ cells per well and incubated overnight at 37 °C with 5% CO_2_. Cells were then transfected with either pLV3-GFP-LC3B (for LC3 puncta detection) or pLV3-mCherry-GFP-LC3B (for autophagic flux detection) using Lipofectamine 3000 reagent according to the manufacturer’s instructions. After 16 h, the medium was replaced with a fresh DMEM medium supplemented with 10% FBS and 1% antibiotics. Cells were co-treated with PG (0.5 μg/mL) or TMZ (800 μM for T98G and 600 μM for A172, respectively) for 48 h. Following treatment, the cells were washed with PBS, fixed with 4% paraformaldehyde for 10 min, stained with DAPI (Beyotime, China) for 5 min, and finally were observed under a confocal microscope (LSM 900, Zeiss, Oberkochen, Baden-Württemberg, Germany).

For paxillin staining, T98G and A172 cells grown on coverslips after PG or TMZ treatment were fixed in 4% paraformaldehyde for 15 min at room temperature, permeabilized with 0.1% Triton X-100 in PBS for 15 min at room temperature, then blocked with 1% BSA in PBS for 1 h at room temperature. Cells were then incubated with Rabbit anti-Paxillin antibody (HUABIO, Hangzhou, China) at 1/50 dilution in 1% BSA in PBST overnight at 4 °C. Alexa Fluor^®^ 555-labeled Goat Anti-Rabbit IgG H&L (abcam, Cambridge, UK) was used as the secondary antibody at a 1/500 dilution in PBST, incubated for 1.5 h in the dark. Subsequently, actin filaments were stained with Alexa Fluor^®^ 488-conjugated phalloidin (Beyotime, Shanghai, China) at a 1/200 dilution in PBS for 30 min, protected from light. Nuclear DNA was counterstained blue with DAPI (Beyotime, Shanghai, China). Images were captured using a confocal microscope (LSM 900, Zeiss, Oberkochen, Baden-Württemberg, Germany) equipped with appropriate filter sets. For the quantification of the FA number, background subtraction from selected images was performed as described [31].

### 2.10. RNA Sequencing and Bioinformatic Analyses

T98G cells were seeded at 850,000 per well in 10 cm dishes and incubated overnight at 37 °C with 5% CO_2_. The next day, cells were treated with 0.5 μg/mL PG with or without 800 μM TMZ for 24 h, and then cell pellets were harvested. Total RNA was extracted with Trizol reagent (Invitrogen, Carlsbad, CA, USA). RNA integrity and quantification were assessed by the Bioanalyzer 2100 system (Agilent Technologies, Santa Clara, CA, USA). Library preparation and RNA sequencing were conducted on the platform of Illumina HiSeq 6000 in Novogene Bioinformatics Technology Co., Ltd. (Beijing, China).

Raw sequencing reads in fastq format were quality-controlled and trimmed using fastp v1.0.1 software. Clean data was then aligned with reference human hg38 sequence with HISAT2 (2.2.1). Read counts mapped to each gene were quantified using FeatureCounts (version 2.0.6). FPKM of each gene was calculated based on the length of the gene and reads count mapped to this gene. Differential expression analysis (DEGs) for two groups was performed using the DESeq2 R package 3 (1.42.0) according to the criteria of |log2(fold change)| ≥ 1 and *p*-adjust  ≤ 0.05. The DEGs were clustered into distinct groups using the Mfuzz or K-means algorithm using Weishengxin “https://www.bioinformatics.com.cn/” (accessed on 22 December 2025) Subsequently, the statistical enrichment of down-regulated DEGs in KEGG pathways was conducted by Metascape v3.5.

### 2.11. Statistical Analysis

The data are expressed as mean ± SD and were analyzed using GraphPad Prism 9. One-way analysis of variance (ANOVA) was used to analyze differences between groups, followed by a post hoc (Bonferroni) test. A *p*-value of <0.05 was considered statistically significant.

## 3. Results

### 3.1. PG and TMZ Synergistcially Inhibited the Proliferation of Glioblastoma Cells

**Figure 1:**
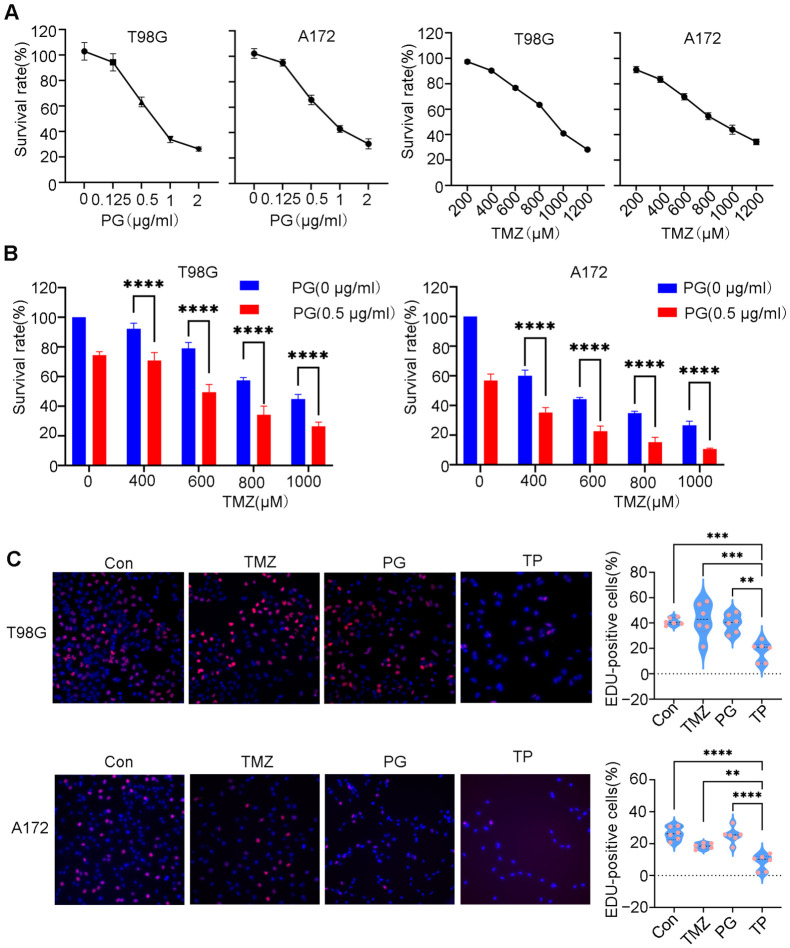
Prodigiosin suppresses the proliferation of glioma cells and enhances their sensitivity to TMZ. (**A**) Cell viability was measured using a CCK8 assay after treating T98G and A172 cells with different doses of prodigiosin (PG) or TMZ for 48 h. *n* = 3. (**B**) T98G and A172 cells were treated for 48 h with range doses of TMZ (400, 600, 800, 1000 μM) in the presence or absence of PG (0.5 μg/mL), followed by cell viability assessment using a CCK8 assay. *n* = 3. (**C**) The proliferation of T98G and A172 cells treated with PG (0.5 μg/mL) or TMZ (800 μM and 600 μM, respectively) was measured using the EDU assay (EDU, red; DAPI, blue). *n* = 6. TP means the combination of TMZ and PG. ** *p* < 0.01, *** *p* < 0.001, **** *p* < 0.0001.

One study has shown that PG inhibited proliferation in glioblastoma cells [32]. The two human glioblastoma cell lines, T98G and A172, were treated with 0, 0.125, 0.25, 0.5,1.0, 2.0 μg/mL PG or 200, 400, 600, 800, 1000, 1200 μM TMZ for 48 h. Then cell viability was examined by a CCK8 assay. The result showed that PG showed an inhibitory effect on the viability of T98G and A172 cells in a dose-dependent manner (Figure 1A), with IC50 values of 0.47 µg/mL and 0.58 µg/mL, respectively. Based on these results, 0.5 µg/mL (the average IC50 of the two cell lines) was selected for subsequent combination experiments with TMZ, as this concentration provides a consistent ~50% inhibition in both cell lines. Similarly, the inhibitory effect of TMZ on T87G and A172 cell proliferation was concentration-dependent (Figure 1A). Targeting the autophagy pathway represents a promising strategy of increasing TMZ sensitivity to glioblastoma cells [33]. To analyze whether PG sensitize tumor cells to TMZ, T98G and A172 cells were treated with 0.5 μg/mL PG in combination with increasing concentrations of TMZ. The combination treatment significantly enhanced cell inhibition compared to TMZ alone (Figure 1B). Likewise, the combined therapy (TP) showed a greater fraction of EDU-positive cells than the corresponding single drug in the both cells (Figure 1C). These findings demonstrate that PG potentiated the anti-proliferative effect of TMZ, leading to significantly reduced proliferation of glioblastoma cells.

### 3.2. PG Synergizes with TMZ to Induce Tumor Cell Apoptosis

**Figure 2:**
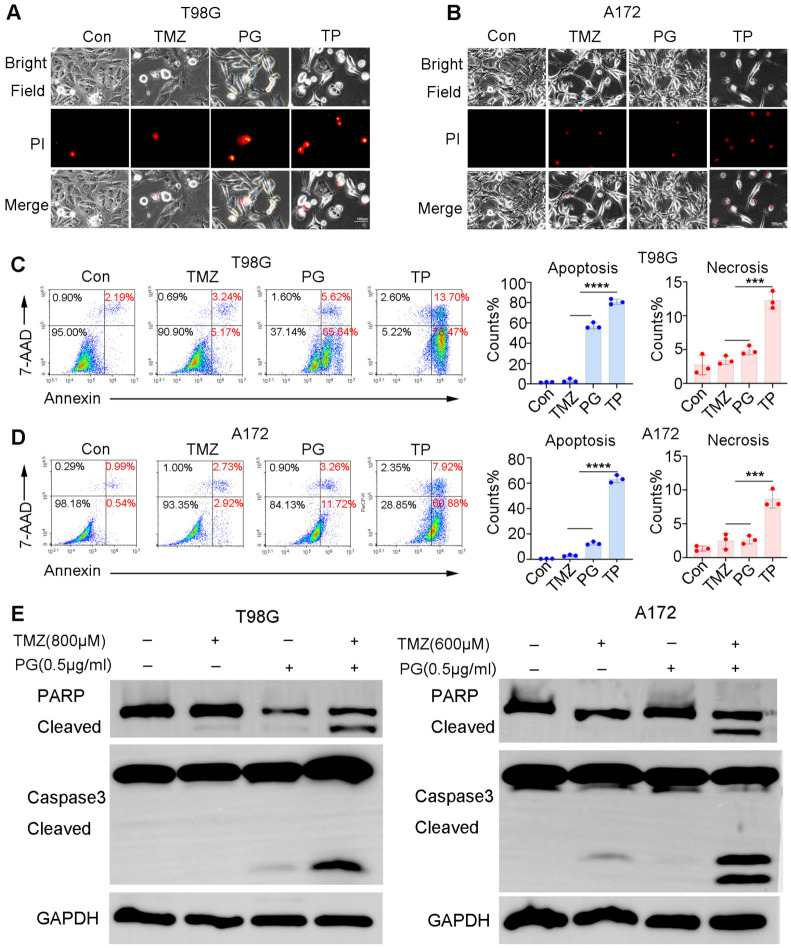
The combination of PG and TMZ induced the apoptosis of glioma cells. (**A**,**B**) The T98G and A172 cells were cotreated with PG (0.5 μg/mL) or TMZ (800 μM and 600 μM) for 48 h, respectively. The apoptotic cells were stained with 2 μg/mL PI for fluorescence microscopy imaging (**A**,**B**) or Annexin V/7-AAD for flow cytometry. (**C**,**D**) Representative microscopy images of T98G (**C**) and A172 (**D**) cells are shown (Scale bar = 100 μm). (**E**) The cell lysates were collected and analyzed via Western blotting using antibodies against PARP and caspase3. GAPDH served as the loading control. TP means the combination of TMZ and PG. *** *p* < 0.001, **** *p* < 0.0001. The original Western blot image is presented in Supplementary Materials, Figure S4.

To assess the apoptotic effect of the PG and TMZ combination on glioblastoma cells, the T98G and A172 cells were treated with 0.5 μg/mL PG in combination with TMZ (at 800 μM and 600 μM) for 48 h, respectively. Firstly, a Propidium Iodide (PI) staining was performed by fluorescence microscopy analysis. As a membrane-impermeable dye, PI selectively enters apoptotic or necrotic cells and binds to DNA, thereby emitting red fluorescence [34]. As shown in Figure 2A,B, the combination treatment led to a marked decrease in the number of T98G and A172 cells in bright field, corresponding to the anti-proliferative effect. Similarly, PG and TMZ resulted in an increase in PI-derived red fluorescence. Consistent with this, the combined therapy markedly increased apoptosis and necrosis in the T98G and A172 cells by flow cytometry with Annexin V/7-AAD staining (Figure 2C,D). Cleaved caspase-3 and cleaved poly(ADP-ribose) polymerase (PARP) were two widely used apoptotic markers [35]. Furthermore, Western blot results showed that the combination of PG and TMZ enhanced the levels of cleaved caspase-3 and PARP compared to PG and TMZ monotherapy (Figure 2E and Figure S4). These findings highlight the higher levels of apoptosis and necrosis induced by PG or TMZ combination therapy.

### 3.3. PG Inhibits Migration and Invasion of Glioblastoma Cells When Combined with TMZ

**Figure 3:**
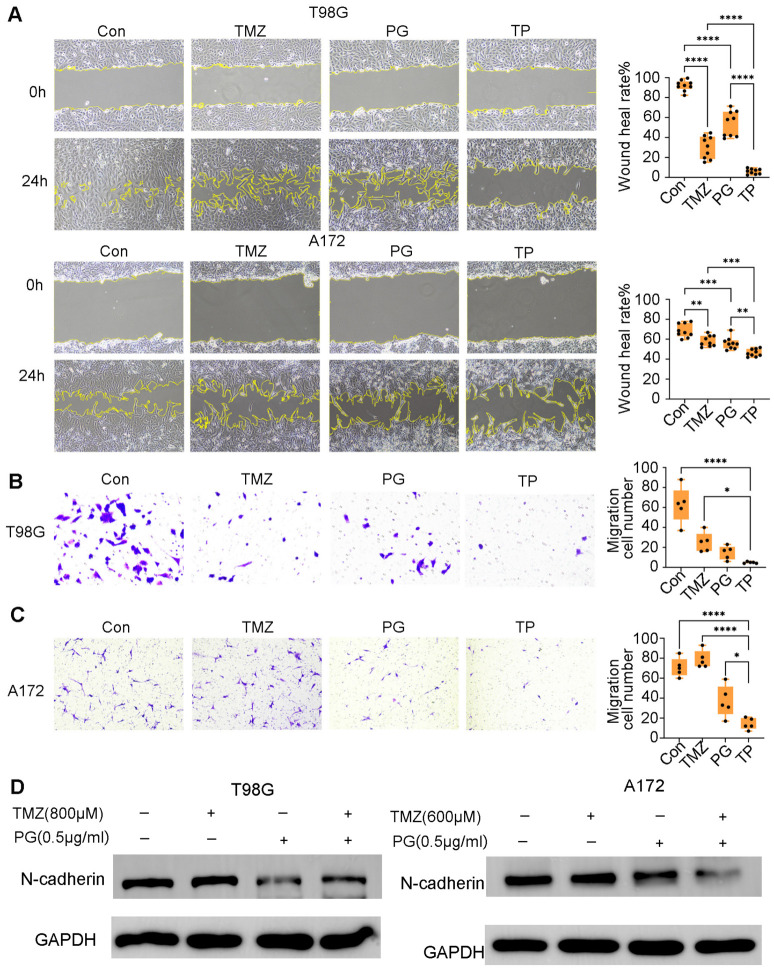
The combination of PG and TMZ inhibited migration and invasion of glioma cells. (**A**) Wound healing assay showing migration of T98G and A172 cells after treatment with PG (0.5 μg/mL) or TMZ (800 μM and 600 μM) for 24 h, respectively. Yellow lines indicate the wound edges, and the area between the yellow lines represents the residual wound gap Scale bar = 100 μm. (**B**,**C**) Representative images of transwell assays demonstrating the invasion of T98G and A172 (**C**) cells following 24 h treatment with PG (0.5 μg/mL) or TMZ (800 μM and 600 μM), respectively. (**D**) Western blot shows that TP decreased N-cadherin expression at 48 h. TP means the combination of TMZ and PG. * *p* < 0.05, ** *p* < 0.01, *** *p* < 0.001, **** *p* < 0.0001. The original Western blot image is presented in Supplementary Materials, Figure S5.

To investigate the effect of the drug treatment on cellular migratory and invasive capabilities, T98G and A172 cells were treated with 0.5 μg/mL PG in combination with TMZ (at 800 μM and 600 μM) for 24 h, respectively. The 24 h treatment duration was selected because prolonged incubation would lead to substantial cell death due to the cytotoxic effects of the combination treatment, which could confound the assessment of migration. As shown in the wound healing assay (Figure 3A), in both T98G and A172 cells, TMZ or PG alone significantly inhibited wound closure compared to the Con group. Notably, the combination of PG and TMZ further enhanced this inhibitory effect, resulting in an even greater suppression of wound healing compared to either monotherapy. Similarly, the transwell assay demonstrated that the combination treatment significantly inhibited A172 cell invasion as compared to monotherapy (Figure 3C), while in T98G cells, it showed an enhanced but statistically non-significant inhibitory effect as compared to PG alone (Figure 3B). The cell–cell adhesion molecule N-cadherin is associated with increased mortality and poor overall survival in glioblastomas [36]. Western blot analysis of T98G and A172 cell lysates revealed a decrease in the expression of N-cadherin after treatment for 48 h (Figure 3D and Figure S5). These findings suggest that the combination of PG and TMZ could suppress cellular migratory and invasive capabilities of glioblastoma cells.

### 3.4. Functional Enrichment Analysis of DEGs from RNA-Seq After PG-TMZ Treatment

**Figure 4:**
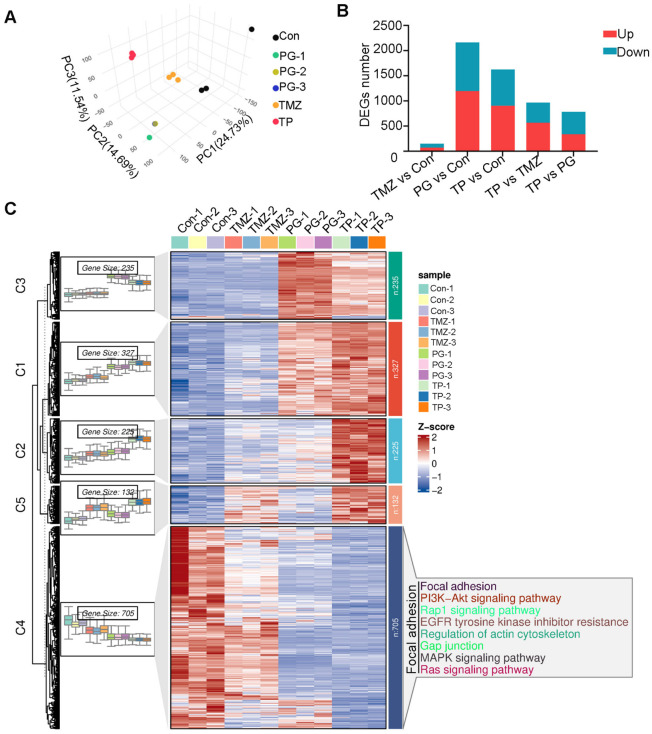
PG in combination with TMZ modulated Focal adhesion signaling. (**A**) 3D visualization of principal component analysis (PCA). In the 3D plot, PG-2 and PG-3 largely overlapped with each other. (**B**) Bar chart of differentially expressed genes between groups. (**C**) The KEGG analyses of differentially expressed genes, identifying pathways associated with Focal adhesion signaling. TP means the combination of TMZ and PG.

In order to further explore the potential mechanism alterations of the PG-TMZ combination on T98G cells, the cells were treated with 0.5 μg/mL PG in combination with 800 μM TMZ for 24 h, and then RNA-Seq was performed. The clear separation of all groups in the PCA plot (Figure 4A) demonstrates significant global transcriptomic differences resulting from PG and/or treatment. Furthermore, differential genes (DEGs) were detected in accordance with the strict criteria of |log2FC| ≥ 1 and *p* ≤ 0.05. TMZ produced limited transcriptional changes in T98G cells compared to the Con group, PG induced broad alterations. A total of 907 genes were upregulated and 721 genes in the combination treatment were downregulated relative to the Con group (Figure 4B). DEGs identified from the combination therapy compared to Con groups were subsequently clustered using the Mfuzz/K-means algorithm. The downregulated genes were optimally grouped into a single cluster and then conducted to the KEGG analysis by Metascape. The results showed that downregulated genes were primarily enriched in focal adhesion signaling, comprising multiple secondary pathways such as Focal adhesion, the PI3K-Akt signaling pathway, the ap1 signaling pathway, EGFR tyrosine kinase inhibitor resistance, regulation of actin cytoskeleton, gap junction, the MAPK signaling pathway and Ras signaling pathway (Figure 4C).

### 3.5. Combinational Treatment of PG and TMZ Increased FA Number and Altered AKT Phosphorylation

**Figure 5:**
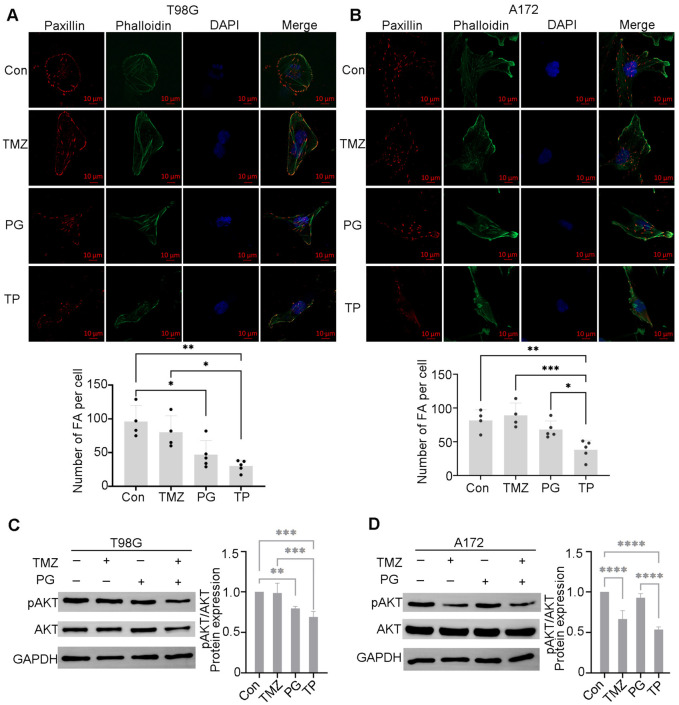
Co-treatment with PG and TMZ decreased focal adhesion number and AKT phosphorylation. (**A**,**B**) Immunofluorescent confocal microscopy of focal adhesion (FA) which was stained with Paxillin (red) in T98G (**A**) and A172 cells (**B**). Representative pictures are shown. Nuclei were stained with DAPI (blue) and F-actin with Phalloidin. Scale bar = 10 μm. Focal adhesions per cell were quantified. (**C**,**D**) Western blot shows that TP decreased AKT phosphorylation at 48 h. TP means the combination of TMZ and PG. * *p* < 0.05, ** *p* < 0.01, *** *p* < 0.001, **** *p* < 0. 0001. The original Western blot image is presented in Supplementary Materials, Figure S6.

Focal adhesion(FA)-based cell–extracellular matrix interactions are essential for cell anchoring and cell migration [37]. Based on the suppression of migration and invasion, as well as the attenuation of focal adhesion signaling in the PG-TMZ combination, we decided to evaluate the focal adhesion after the treatment. T98G and A172 cells were treated with 0.5 μg/mL PG in combination with TMZ (at 800 μM and 600 μM) for 48 h, respectively. Cells were co-stained paxillin and F-actin with phalloidin and then the FA per cell was quantified. The results showed a decrease in the average number of focal adhesions per cell after the PG-TMZ treatment in both cells (Figure 5A,B).

Aberrant activation of the PI3K/AKT/mTOR signaling pathway is widely recognized as one of the most common events in cancer. The AKT phosphorylation level of T98G and A172 cells induced by the combination of PG and TMZ was decreased compared to drugs alone (Figure 5C,D and Figure S6).

### 3.6. Combinational Treatment of PG and TMZ Impaired Autophagic Flux

**Figure 6:**
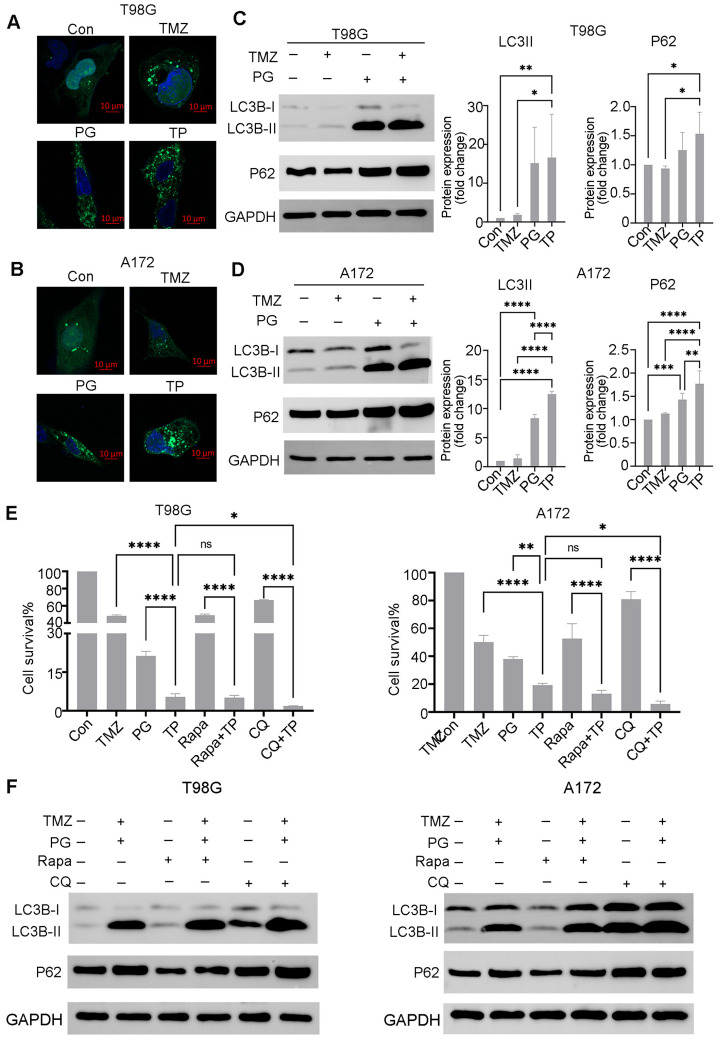
The combination of PG and TMZ blocked autophagic flux. (**A**,**B**) T98G (**A**) and A172 (**B**) cells expressing GFP-LC3 fluorescence were treated with PG or TMZ for 48 h. Scale bar = 10 μm. (**C**,**D**) Western blot showed that TP increased the level of LC3 and P62 expression in T98G (**C**) and A172 (**D**) cells at 48 h. (**E**,**F**) T98G and A172 cells were treated with the combination of PG and TMZ for 48 h in presence or absence of Rapamycin (Rapa) or CQ. CCK8 assay was used to determine cell viability. (**E**) *n* = 3. The level of LC3 and P62 expression was analyzed by Western blot. (**F**) TP means the combination of TMZ and PG. * *p* < 0.05, ** *p* < 0.01, *** *p* < 0.001, **** *p* < 0. 0001. The original Western blot image is presented in Supplementary Materials, Figure S7.

Multiple studies have shown that PG inhibits autophagy [38,39]. To examine the correlation between PG-TMZ combination and autophagy, T98G and A172 cells expressing GFP-LC3 were assessed for autophagosome formation. The findings showed that GFP-LC3 spots were significantly increased after the treatment with PG alone or the combination of PG and TMZ (Figure 6A,B). P62, which serves as a receptor for ubiquitinated protein aggregates, is selectively degraded via autophagy. Impaired autophagy leads to P62 accumulation [40]. TMZ treatment had a minimal effect on the expression levels of LC-3II and P62. However, both LC3II and P62 increased significantly following PG treatment. Furthermore, the combination of PG and TMZ led to a more pronounced increase in LC3II and P62 compared to PG alone in A172 cells (Figure 6D). In contrast, while the combination resulted in a greater increase than PG alone in T98G cells, the difference was not statistically significant (Figure 6C). Since P62 levels were induced after TP treatment, we hypothesized that TP might impair autophagic flux.

To further investigate the effect of TP treatment on autophagic flux, T98G and A172 cells were transfected with the pLV3-mCherry-GFP-LC3B plasmid. Under normal autophagic flux, autophagosomes fuse with lysosomes to form autolysosomes; the acidic lysosomal environment quenches GFP fluorescence while mCherry remains stable, enabling autolysosomes to be visualized as red puncta. Conversely, when the autophagosome–lysosome fusion is blocked or lysosomal acidification is impaired, yellow puncta accumulate. In the control and TMZ groups, mCherry-GFP-LC3B fluorescence was predominantly diffused throughout the cytoplasm, with minimal puncta formation. In contrast, treatment with PG alone or TP led to the accumulation of yellow puncta, similar to the positive control chloroquine (CQ). These results indicate that TP treatment impairs autophagic flux, likely by blocking autophagosome–lysosome fusion or inhibiting lysosomal acidification (Figures S1 and S2).

Rapamycin (Rapa) can activate autophagy by inhibiting mTOR signaling; chloroquine (CQ) is an inhibitor of the late stage of autophagy, inhibiting lysosomal acidification and preventing autophagosome degradation. Bafilomycin A1 (Baf A1) is a specific inhibitor of vacuolar-type H^+^-ATPase (V-ATPase), blocking lysosomal acidification and thereby preventing autophagosome–lysosome fusion and autophagosomal degradation. The CCK-8 assay showed that Rapa had almost no effect on TP-induced inhibition of cell proliferation and cytotoxicity (Figure 6E). In contrast, both CQ and Baf A1 significantly enhanced the anti-proliferative and cytotoxic effects of TP treatment, with higher concentrations of Bafilomycin A1 yielding greater inhibitory effects (Figure 6E and Figure S3). Correspondingly, Rapa treatment did not affect LC3II/P62 levels in the presence of TP, in contrast to CQ, which induced their further accumulation (Figure 6F and Figure S7). These results suggest that the attenuation of TP’s anti-proliferative and cytotoxic effects on glioblastoma cells was mainly mediated by an autophagy-impairment mechanism.

## 4. Discussion

GBM represents one of the most formidable challenges in oncology due to the high morbidity and mortality rates. While TMZ serves as a first-line treatment for GBM, the frequent emergence of resistance becomes a major factor in effective treatment. Therefore, it is urgent to develop new compounds to overcome this limitation. The study investigated if PG, as a chemosensitizer, can increase the chemosensitivity of GBM cells to TMZ by significantly inhibiting GBM cell viability, inducing apoptosis, suppressing focal adhesion signaling and impairing autophagic flux. These findings suggest its potential as an adjuvant therapeutic agent for TMZ-based treatment strategies.

PG, as a natural secondary metabolite extracted from *Serratia marcescens* and other bacteria, has attracted much attention due to its anti-tumor activity [21]. Studies have reported that PG significantly inhibits the proliferation and reduced neurosphere formation ability of GBM cells [26,32]. In the present study, we demonstrated that PG exhibited a dose-dependent inhibitory effect on the proliferation of T98G and A172 cells. PG also displayed potential chemosensitizing effects, enhancing the efficacy of drugs as Cisplatin [27] and 5-Fluorouracil (5-Fu) [28] to synergistically inhibit tumor growth. Our results showed that the combination of PG and TMZ significantly reduced proliferation of glioblastoma cells, as confirmed by CCK8 and EDU assays. Apoptosis is the main mechanism for the antitumor activity of PG against several human tumors. Specifically, PG triggered apoptosis through caspase-3 activation and PARP cleavage in GBM cells [26]. Mechanistically, PG alone slightly induced caspase-3 activation and PARP cleavage in T98G and A172 cells, but the combination of PG and TMZ treatment markedly enhanced the effect.

Focal adhesion-mediated adhesion of cells to the extracellular matrix (ECM) is essential for cellular processes, such as cell adhesion, migration, motility, cell survival, and cell proliferation [41]. Focal adhesions are macromolecular complexes consisting of hundreds of interacting proteins including integrins, talin, vinculin, paxillin, alpha-actinin, and integrin-linked kinase [42]. Focal adhesion also critically regulates tumor cell emergence, migration and metastatic progression [37]. Dysregulated focal adhesions in tumor cells altered cell–ECM interactions, enhanced tumor cell motility, and promoted invasive potential [43]. Focal adhesion has been identified as a risk factor associated with chemotherapy efficacy and prognosis in gastric cancer patients; notably, those with a high focal adhesion-related gene score (FAscore) exhibited insensitivity to neoadjuvant chemotherapy [44]. Furthermore, proteins involved in focal adhesion pathways had the potential to be biomarker candidates with GB progression [45]. Therefore, therapeutic strategies targeting focal adhesion signaling or the assemble of focal adhesion structures are increasingly being explored for tumor treatment. For example, the selective focal adhesion kinase (FAK) inhibitor A8 significantly inhibited tumor growth [46]. The knockdown of Integrin α10β1 decreased the migration and survival of GBM cells [47]. Our RNA-seq analysis revealed that the PG-TMZ combination downregulated focal adhesion pathways compared to the Con group, which is consistent with the finding that the combination treatment significantly inhibited cell migration and invasion compared to the Con and TMZ groups, and showed a trend toward further reduction compared to the PG group, although this difference mostly did not reach statistical significance. Paxillin is a focal adhesion protein that can bind to various structural and recruit signaling molecules [48]. Paxillin staining can be employed to visualize focal adhesions complexes. Our results revealed a decrease in the average number of focal adhesions per cell following the combination of PG and TMZ treatment in T98G and A172 cell lines, demonstrating the inhibition of focal adhesion formation. Some studies have reported that disturbing the assembly of new focal adhesion structures could inhibit tumor cell migration [49].

In the KEGG analysis, the down-regulated PI3K-Akt signaling pathway was included in the focal adhesion pathway. The PI3K/AKT pathway is among the most commonly dysregulated signaling cascades in human tumors, a promising therapeutic target for clinical tumor therapy [50,51]. PI3K-PI(3,4,5)P3-AKT signaling can be activated by focal adhesions subcellular signaling hubs in human cancer cells [52]. The overactivation of AKT, the major functional protein in this pathway, has been detected in multiple tumors including colon, gastric, ovarian, pancreatic, oesophageal and hyroid tumors. Recently, capivasertib, as a novel AKT inhibitor, was approved for hormone-receptor-positive, HER-2-negative metastatic breast cancer. Our results show that the combination of PG and TMZ significantly reduced AKT phosphorylation in T98G and A172 cells compared to either drug alone. Studies have also reported that PG inhibited tumor cell growth by suppressing AKT phosphorylation [32,53].

Gradual accumulation of evidence demonstrates that autophagy, a lysosome-dependent degrading route for damaged cellular components, drives the therapeutic resistance in GBM by providing a survival advantage on tumor cells under metabolic, hypoxic, and therapeutic stress conditions [54]. Consequently, targeting autophagy has been highly implicated as a promising therapeutic strategy to overcome drug chemoresistance and radio-resistance in GBM therapy [55]. Studies have reported that PG inhibits tumor cell proliferation or induces apoptosis by inhibiting autophagy or impairs autophagosome–lysosome fusion. Cheng et al. reported a marked increase in LC3 puncta formation along with elevated levels of LC3II in GBM cells following prodigiosin treatment [26]. In our results, the induction of LC3 puncta and expression of LC3II were found in PG-treated T98G and A172 cells. Furthermore, the combination of PG and TMZ triggered a more substantial induction of LC3-II in T98G and A172 cells than PG alone. By functioning as an adaptor that bridges LC3 and ubiquitinated cargo for incorporation into autophagosomes for degradation, p62 is degraded with its cargo in the autolysosome. Therefore, the cellular accumulation of P62 reliably indicates autophagy inhibition or dysfunctional autophagic flux [56]. Our findings show that the pronounced accumulation of p62 was induced by PG and PG-TMZ treatments, implying the blockade of autophagic flux. To confirm the inhibition of that autophagic flux, we employed both an autophagy inducer (Rapamycin, Rapa), an autophagy inhibitor (Chloroquine, CQ) and a late-stage autophagy inhibitor (Bafilomycin A1, Baf A1). Notably, CQ and Baf A1 significantly enhanced the anti-proliferative and cytotoxic effects induced by the combination of PG and TMZ, whereas Rapa showed no such effect. Furthermore, CQ co-treatment led to a further accumulation of both LC3II and p62, which was not observed with Rapa co-treatment. These data collectively indicate that the combination of PG and TMZ exerts anti-proliferative and pro-apoptotic effects primarily through the inhibition of autophagic flux.

Despite the promising efficacy of the PG–TMZ combination therapy observed in vitro, further evaluation in orthotopic animal models, patient-derived xenografts, or GBM organoid systems is required to assess its translational potential. Given that MGMT promoter methylation is a key determinant of TMZ sensitivity [57], integrating MGMT status into future clinical strategies for this combination may enable more precise patient stratification and improved therapeutic outcomes [58]. In parallel, identifying the underlying signaling axis of the PG–TMZ combination by performing experiments—such as AKT reactivation, focal adhesion kinase activation, or restoration of autophagic flux—is essential to close the mechanistic chain. These mechanistic and translational investigations will be prioritized in our follow-up studies.

## 5. Conclusions

In conclusion, our study identifies PG as an effective chemosensitizer that synergistically enhances the anti-GBM efficacy of TMZ. The combined PG-TMZ regimen suppresses tumor cell proliferation and survival through disrupting focal adhesion-mediated pro-survival signaling and impairing cytoprotective autophagic flux. These findings position PG as a promising adjunctive agent in TMZ-based therapy, offering a novel strategic approach to overcome chemoresistance in GBM.

## Data Availability

The original contributions presented in this study are included in the article/Supplementary Material. Further inquiries can be directed to the corresponding author.

## Supplementary Materials

The following supporting information can be downloaded at: https://www.mdpi.com/article/10.3390/biom16070977/s1. Figure S1. Representative fluorescence images of T98G cells trans fected with the pLV3-mCherry-GFP-LC3B. Figure S2. Representative fluorescence images of A172 cells transfected with the pLV3-mCherry-GFP-LC3B. Figure S3. The combination of PG and TMZ blocked autophagic flux. Figure S4. Original Western blot images of PARP, Caspase-3, and GAPDH in A172 and T98G cells in Figure 2E. Figure S5. Original Western blot images of N-cadherin and GAPDH in A172 and T98G cells in Figure 3D. Figure S6. Original Western blot images of pAKT, AKT and GAPDH in A172 and T98G cells in Figure 5C,D. Figure S7. Original Western blot images of LC3B, P62 and GAPDH in A172 and T98G cells in Figure 6C,D,F.
