# Supplementary material for: Prodigiosin Enhanced TMZ Chemosensitivity by Suppressing Focal Adhesion and Inhibiting Autophagy in Glioblastoma Cells
Source: Biomolecules. 2026 Jul 3;16(7):977. doi: 10.3390/biom16070977 (PMC13406636; doi:10.3390/biom16070977)
Supplement: Supplementary file 1 [file biomolecules-16-00977-s001.zip › biomolecules-4305392-supplementary.pdf]

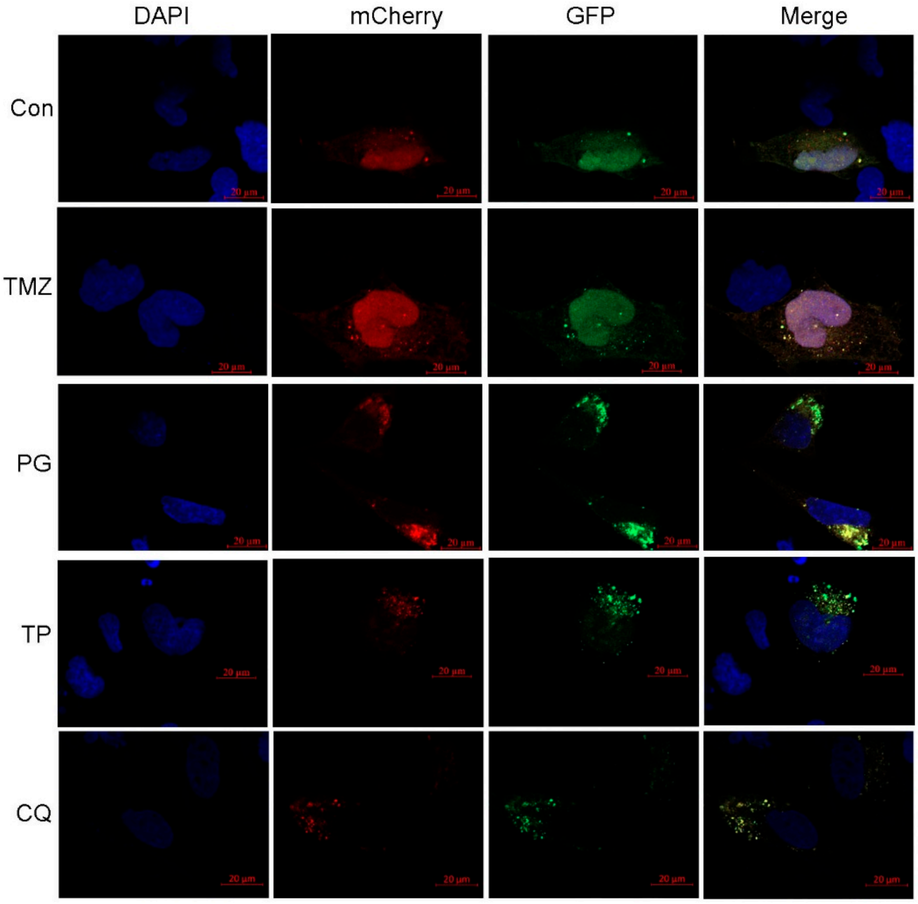

Figure S1. Representative fluorescence images of T98G cells transfected with the pLV3-mCherry-GFP-LC3B. Scale bar = 20 µm.

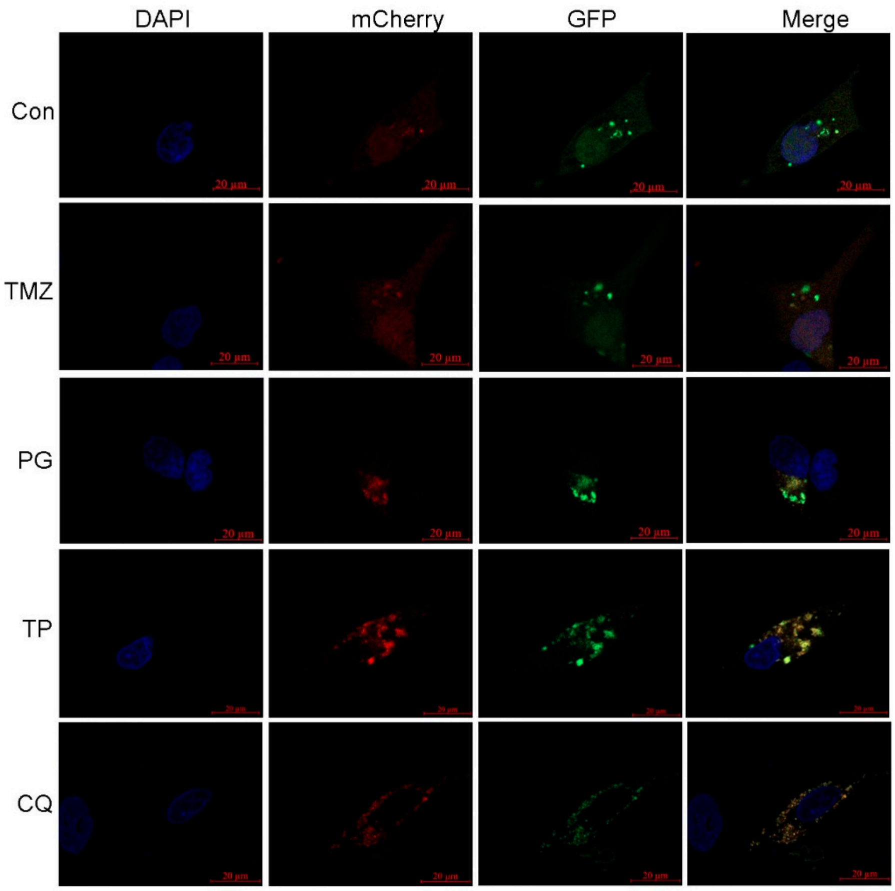

Figure S2. Representative fluorescence images of A172 cells transfected with the pLV3-mCherry-GFP-LC3B. Scale bar = 20 μm.

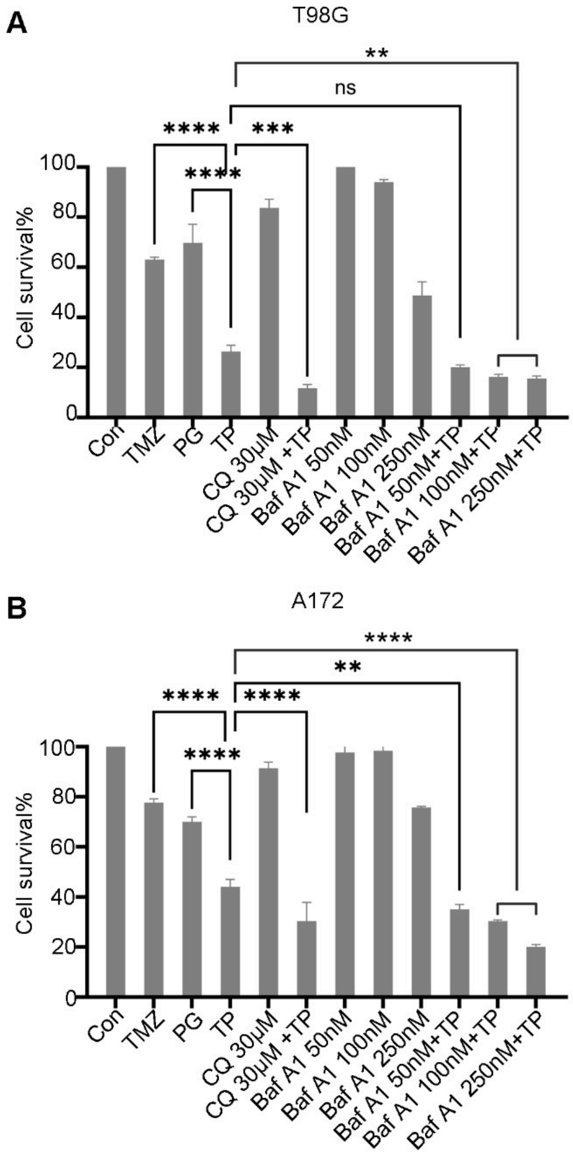

Figure S3. The combination of PG and TMZ blocked autophagic flux**.** A-B， T98G and A172 cells were treated with the combination of PG and TMZ for 48h in presence or absence CQ (30μM) or Baf A1(50, 100, or 250nM). CCK8 assay was used to determine cell viability. TP means the combination TMZ and PG. $**P < 0.01$, $***P < 0.001$, $****P < 0.0001$.

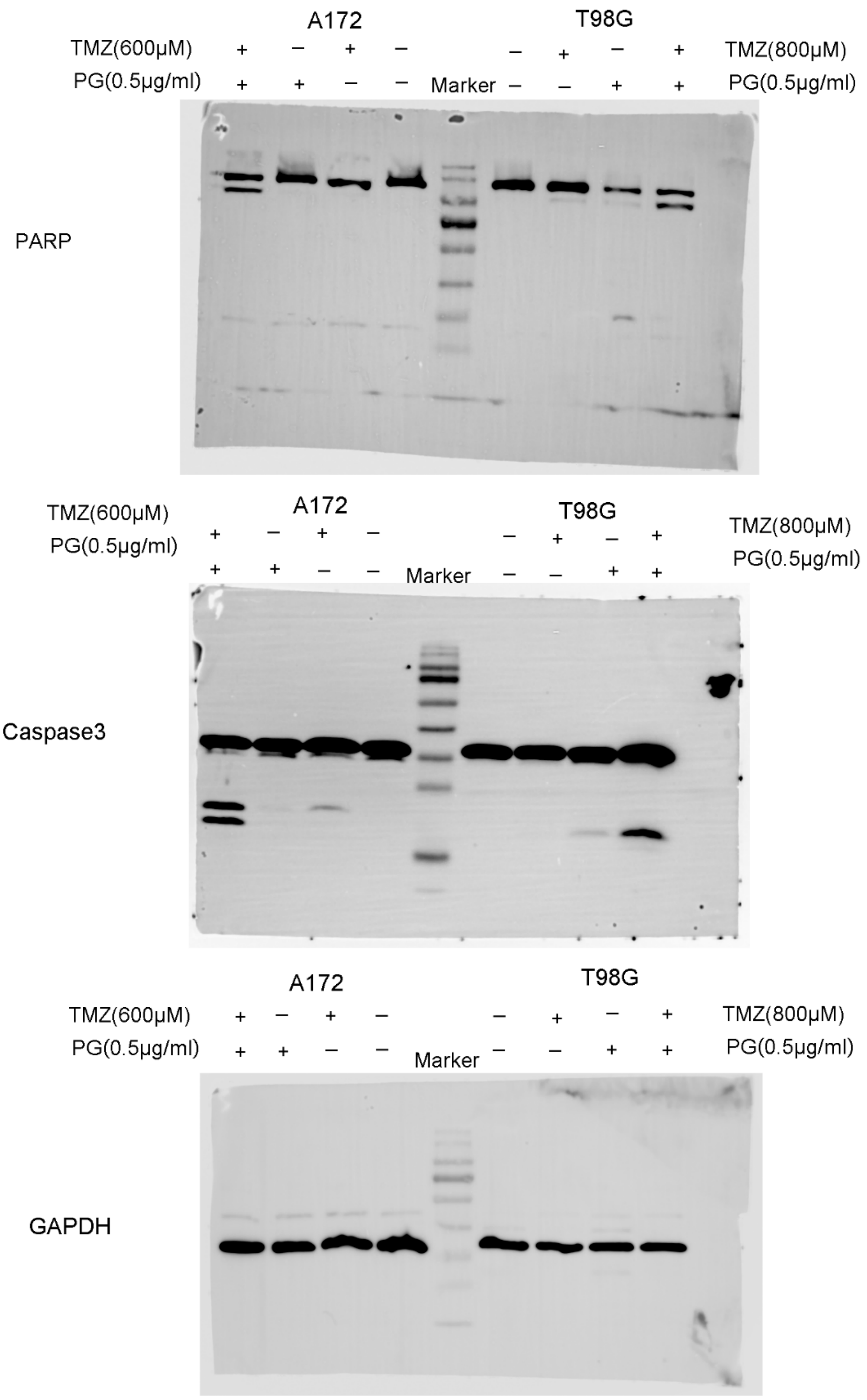

Figure S4. Original Western Blot images of PARP, Caspase-3, and GAPDH in A172 and T98G cells in Figure 2E.

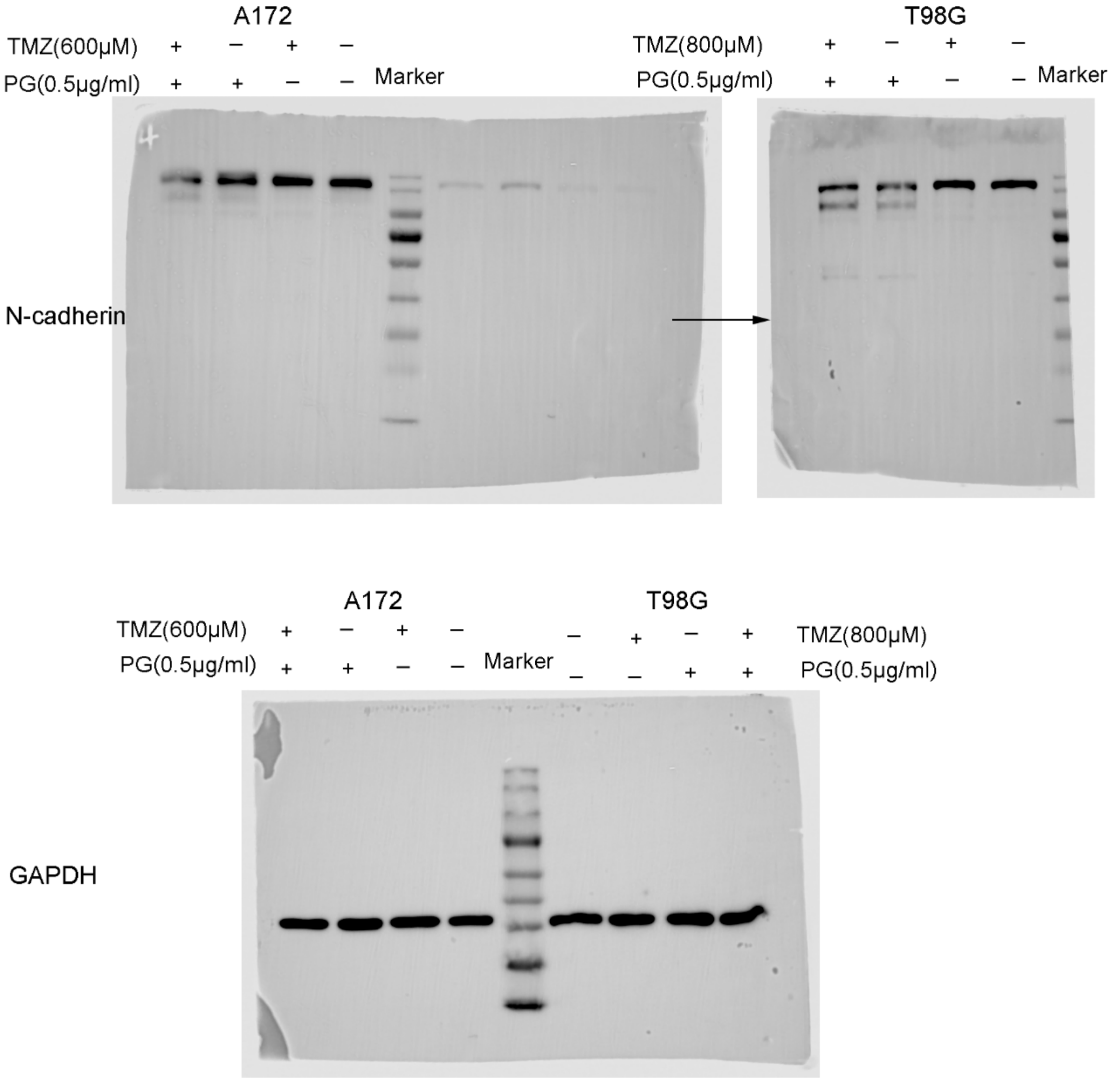

Figure S5. Original Western Blot images of N-cadherin and GAPDH in A172 and T98G cells in Figure 3D.

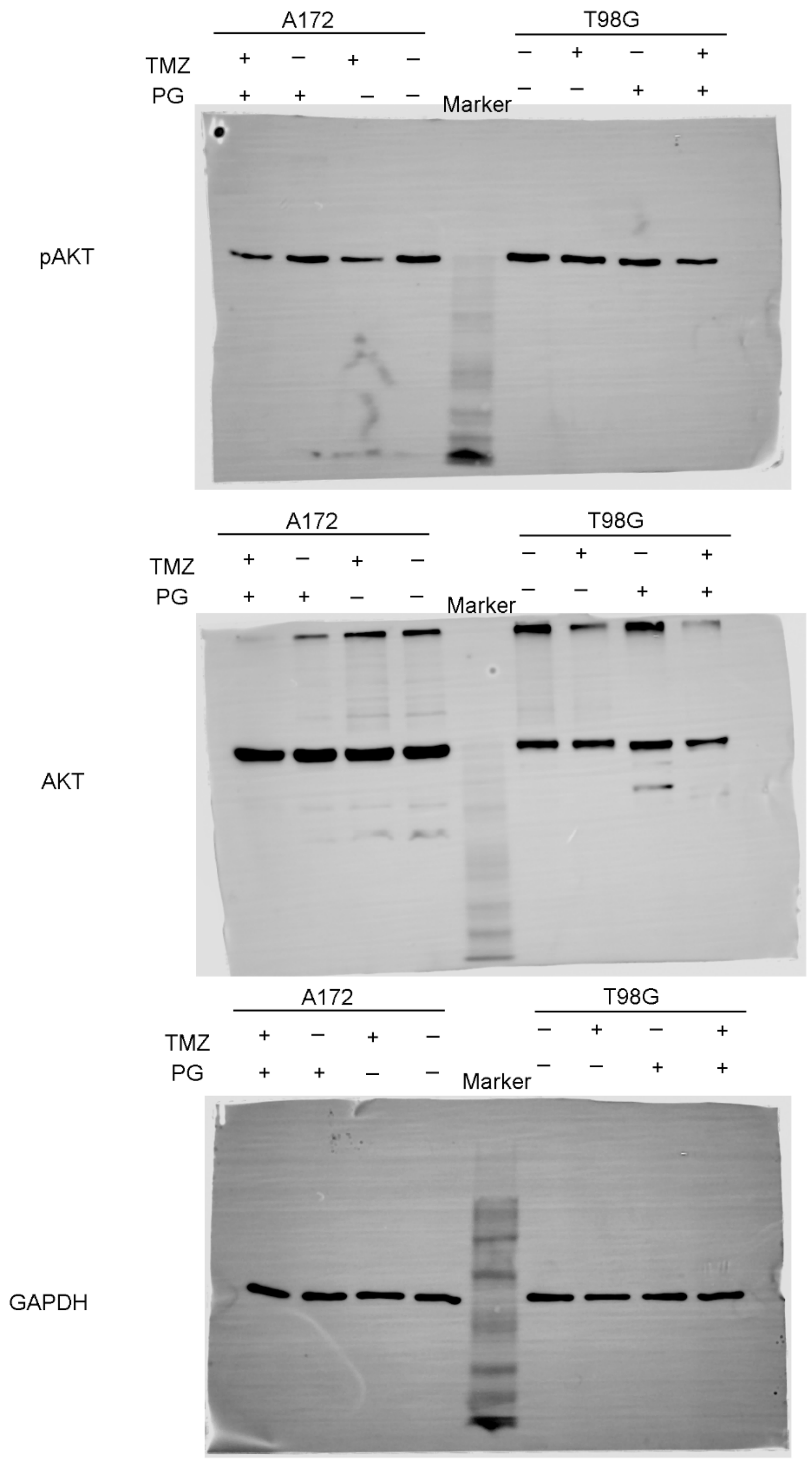

Figure S6. Original Western Blot images of pAKT, AKT and GAPDH in A172 and T98G cells in Figure 5C and 5D.

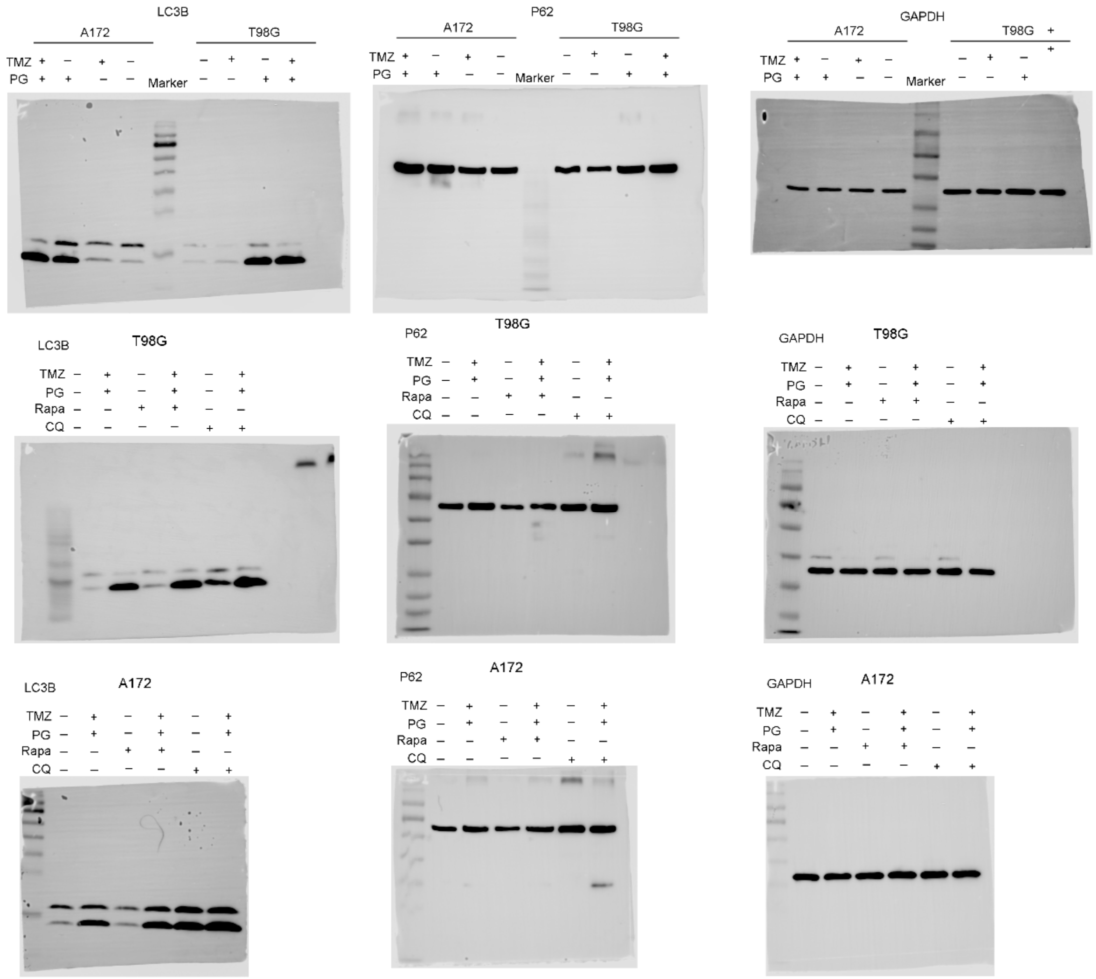

Figure S7. Original Western Blot images of LC3B, P62 and GAPDH in A172 and T98G cells in Figure 6C, 6D and 6F.
